# Grey Matter Changes in Cognitively Impaired Parkinson's Disease Patients

**DOI:** 10.1371/journal.pone.0085595

**Published:** 2014-01-21

**Authors:** Irena Rektorova, Roberta Biundo, Radek Marecek, Luca Weis, Dag Aarsland, Angelo Antonini

**Affiliations:** 1 Brain and Mind Research Program, Central European Institute of Technology, Central European Institute of Technology Masaryk University, Masaryk University, Brno, Czech Republic; 2 First Department of Neurology, School of Medicine, Masaryk University and St. Anne's University Hospital, Brno, Czech Republic; 3 Center for Parkinson's disease and Movement Disorder “Fondazione Ospedale San Camillo” - Istituto di Ricovero e Cura a Carattere Scientifico, Venice-Lido, Italy; 4 Department of Neurology and Clinical Neuroscience, Karolinska Institutet and Karolinska University Hospital, Stockholm, Sweden; 5 Centre for Age-Related Diseases, Stavanger University Hospital, Stavanger, Norway; Philadelphia VA Medical Center, United States of America

## Abstract

**Background:**

Cortical changes associated with cognitive decline in Parkinson's disease (PD) are not fully explored and require investigations with established diagnostic classification criteria.

**Objective:**

We used MRI source-based morphometry to evaluate specific differences in grey matter volume patterns across 4 groups of subjects: healthy controls (HC), PD with normal cognition (PD-NC), PD with mild cognitive impairment (MCI-PD) and PD with dementia (PDD).

**Methods:**

We examined 151 consecutive subjects: 25 HC, 75 PD-NC, 29 MCI-PD, and 22 PDD at an Italian and Czech movement disorder centre. Operational diagnostic criteria were applied to classify MCI-PD and PDD. All structural MRI images were processed together in the Czech centre. The spatial independent component analysis was used to assess group differences of local grey matter volume.

**Results:**

We identified two independent patterns of grey matter volume deviations: a) Reductions in the hippocampus and temporal lobes; b) Decreases in fronto-parietal regions and increases in the midbrain/cerebellum. Both patterns differentiated PDD from all other groups and correlated with visuospatial deficits and letter verbal fluency, respectively. Only the second pattern additionally differentiated PD-NC from HC.

**Conclusion:**

Grey matter changes in PDD involve areas associated with Alzheimer-like pathology while fronto-parietal abnormalities are possibly an early marker of PD cognitive decline. These findings are consistent with a non-linear cognitive progression in PD.

## Introduction

Dementia is highly prevalent in the advanced stages of Parkinson's disease (PD) with important consequences for quality of life of affected patients and caregivers. Imaging evidence suggests an association with discrete regional grey matter abnormalities, but the extent and topographical distribution of such changes at initial stage of cognitive decline are still unknown. Understanding the underlying mechanism of the early cognitive changes in PD is important for diagnosis and prognosis, and ultimately for developing drug treatment to slow progression to dementia [1]. Already at mild cognitive impairment (MCI) level, there is involvement of multiple cognitive domains [2], indicating that several cortical regions are involved. Although some authors have reported cortical atrophy already in MCI-PD [3], other recent voxel-based morphometry (VBM) studies did not find any significant grey matter changes in MCI-PD patients as compared to PD with normal cognition [4]–[6]. Of note however, most MRI studies have not employed the new MCI-PD Movement Disorder Society (MDS) criteria that were developed to avoid shortcomings in cognitive testing and usage of variable diagnostic criteria [7].

MRI analysis methods may also contribute to the results inconsistency. The source based morphometry (SBM) is a newly established multivariate technique that has been shown to be superior to mass-univariate methods using either voxel or cluster level of inference in the identification of specific between-group grey matter changes [8], [9]. The method is based on identification of independent patterns in grey matter images and subsequent statistical analysis based on comparing patterns' expression in individual groups.

In order to shed further light on the specific grey matter volume (GMV) changes in well-defined cognitively impaired subjects with PD, we used the SBM analysis and examined a relatively large sample of 126 PD patients including PD with normal cognition (PD-NC), MCI-PD and PD with dementia (PDD) groups using recent MDS criteria. Findings were compared with cognitive functioning based on a comprehensive battery of tests.

## Subjects and Methods

We examined altogether 151 participants, including 25 HC, 75 PD-NC, 29 MCI-PD, and 22 PDD at two movement disorder centres: an Italian centre (Parkinson's disease Unit of the ‘San Camillo’ Hospital, Venice Lido, Italy) and one Czech centre (First Department of Neurology, St. Anne's Teaching Hospital and CEITEC MU, Masaryk University, Brno). A series of 89 consecutive PD patients (52 PD-NC, 26 PD-MCI and 11 PDD) and 10 HC were recruited in the Italian Centre. In the Czech Centre, 37 PD patients (23 PD-NC, 11 PDD, and 3 MCI-PD) and 15 HC were consecutively enrolled. All PD patients were diagnosed with idiopathic PD according to UK Brain Bank criteria [10] by a movement disorders' specialist. Demographic data and neurological details are summarized in Table 1. Individuals with significant cardiovascular problems, history of major psychiatric disorders, history of stroke and ischaemia focus and/or extensive white matter lesions on brain imaging were excluded. Only those subjects who had the capacity to consent were enrolled.

**Table 1:** Demographic and Clinical data.

		HC	PD NC	PD MCI	PDD
**Age**	Years	**57.8** (53.1÷65.5)	**64.2** (53.7÷70.5)	**67.0** (59.5÷70.6)	**70.7** (64.1÷73.9)
**Gender**	males/females	**13/12**	**43/32**	**19/10**	**14/8**
**Education**	Years	**13** (12÷17)	**13** (8÷17)	**8** (7÷13)	**10** (8÷13)
**C1**		**0.43** (−0.25÷0.83)	**−0.01** (−0.63÷0.55)	**0.30** (−0.03÷0.63)	**−0.55** (−1.59÷0.14)
**C2**		**0.52** (−0.05÷1.02)	**0.07** (−0.28÷0.63)	**0.12** (−0.41÷0.77)	**−0.16** (−1.46÷0.11)
**ICV**	cubic dm	**1.15** (1.02÷1.22)	**1.18** (1.03÷1.24)	**1.06** (1.03÷1.19)	**1.06** (0.91÷1.11)
**MMSE**		**30** (29÷30)	**29** (28÷30)	**28** (26÷28)	**24** (20÷26)
**Disease Duration**	Years	*	**5** (3÷9)	**9** (6÷11)	**5** (2÷10)
**H-Y Stage**		*	**2.0** (1.5÷3.0)	**3.0** (2.0÷3.0)	**3.0** (2.0÷3.0)
**LED**	mg/day	*	**647** (370÷910)	**824** (605÷1210)	**775** (510÷1064)

* not applicable; data are given as median (lower quartil÷upper quartil); C1: component loading; C2: component loading; MMSE: Mini Mental State Examination; H-Y Stage: Hoehn & Yahr score (Fahn et al. 1987) [49]; LED: levodopa equivalent dose calculated according to Tomlinson et al. 2010 [50].

The study was approved by the ethics committee of the IRCCS San Camillo, Venice, Italy and the local ethics committee in Brno, Czech Republic. Written informed consent was obtained from all participants.

### Neuropsychological examination

At the Italian centre, all participants performed a comprehensive neuropsychological evaluation including assessment of attention/working memory, executive functions, memory, language and visuo-spatial functions (for individual tests and references, see Table 2).

**Table 2:** Cognitive outcomes.

ITALIAN CENTRE	HC	PD NC	PD MCI	PDD
**Attention (z score)**				
**Trail Making Test A ** [51]	**1.03**±0.383	**0.85**±0.685	**−0.68**±2.205	**−3.75**±3.147
**Trail Making Test B ** [51]	**0.96**±0.341	**0.28**±1.458	**−1.57**±1.61	**−1.32**±1.171
**Trail making Test B-A ** [51]	**0.77**±0.35	**0.11**±1.817	**−1.78**±2.044	**−0.64**±1.266
**Stroop Color/Word Test (time interference effect) ** [52]	**0.19**±1.373	**−0.41**±1.088	**−1.56**±2.056	**−4.62**±7.442
**Stroop Color/Word Test (error Interference effect) ** [52]	**0.49**±0.195	**0.27**±0.571	**−1.39**±2.664	**−0.70**±1.19
**Esecutive Functions/working memory (z score)**				
**Frontal Assessment Battery ** [53]	**−0.59**±1.614	**−0.33**±1.348	**−2.38**±2.376	**−3.55**±2.665
**Digit Span Forward ** [54]	**0.43**±1.388	**0.58**±1.315	**0.19**±1.273	**−0.86**±0.759
**Corsi's Test ** [54]	**1.51**±1.629	**0.48**±1.108	**−0.06**±1.11	**−0.2**±0.693
**Memory (z score)**				
**Rey-Osterrieth Complex Figure Test-delayed recall ** [55]	**0.49**±0.933	**−0.05**±0.777	**−0.71**±0.794	**−1.16**±0.683
**Rey Verbal Learning Test-immediate recall ** [54]	**0.78**±1.966	**0.47**±1.475	**−0.95**±1.422	**−2.5**±0.524
**Rey Verbal Learning Test -delayed recall ** [54]	**0.32**±1.312	**0.24**±1.282	**−1.54**±1.248	**−2.16**±0.896
**Visual-spatial functions (z score)**				
**Rey-Osterrieth Complex Figure Test (copy) ** [55]	**0.93**±0.35	**0.03**±1.157	**−1.72**±1.492	**−2.93**±2.116
**Drawing Coping Test ** [56]	**0.70**±0.339	**0.09**±0.696	**−0.76**±1.26	**−4.45**±3.073
**Language (z score)**				
**Letter fluency task ** [56]	**0.81**±1.169	**0.77**±1.302	**−0.18**±0.968	**−0.95**±1.076
**Category Fluency task ** [56]	**1.61**±1.613	**0.74**±1.223	**−0.32**±1.072	**−1.6**±1.483

CZECH CENTRE	HC	PD NC	PD MCI	PDD
**ACE-R raw score**	**95.1**±2.95	**93.4**±3.61	**82.7**±7.57	**70.9**±10.6
**ACE-R Z-score**	**0.00**±1.00	**−0.60**±1.22	**−4.23**±2.568	−**8.22**±3.610
**Attention**	**0.30**±0.492	**0.43**±0.394	**−1.20**±1.643	**−3.12**±2.116
**Memory**	**0.58**±0.615	**0.20**±1.10	**−2.14**±2.454	**−2.88**±1.267
**Executive Functions**	**0.38**±1.088	**0.13**±1.03	**−0.79**±0.955	**−2.87**±1.790
**Language**	**0.41**±0.356	**0.07**±0.449	**−1.29**±2.230	−**2.73**±2.717
**Visuo-spatial Functions**	**0.44**±0.733	**0.40**±0.580	**−0.47**±1.235	**−2.60**±2.073

Data are given as mean ± standard deviation.

At the Czech centre, all subjects underwent the Addenbrooke's Cognitive Examination - Revised (ACE-R) [11], [12]. ACE-R is a relatively detailed screening instrument for dementia [11]. It consists of 18 tasks structured into 5 cognitive domains evaluating memory, verbal fluency, attention and orientation, speech, and visual–spatial abilities. The test includes the mini-mental state examination (MMSE), the result of which can be extracted from the total scores. The maximum score of ACE-R is 100 points (i.e. the best cognitive performance) and the minimum is 0 (i.e. the worst cognitive performance). According to recent studies ACE-R is a valid tool for PD cognitive evaluation [13]–[15] displaying good correlation with both scales specifically designed for cognitive deficits in PD, such as SCOPA-COG, as well as with less specific tests such as MMSE. We used ACE-R for cognitive evaluation also in our previous MRI studies focusing on resting state networks in PD [16], [17]. Using a cut-off <89 points McColgan et al. [18] have shown that ACE-R is useful also for screening MCI-PD. In addition to cut-off score we calculated relevant z-scores for the whole ACE-R and for each of 5 ACE-R cognitive domains based on the data collected from 100 healthy older controls [12]. In the second step, patients with no significant impairment of activities of daily living due to cognitive impairment (i.e. did not have dementia) but achieving <89 points on ACE-R and/or performing ≤2 z-score below the calculated mean in at least one of five ACE-R cognitive domains (i.e. PD-MCI) underwent neurocognitive testing using a comprehensive cognitive battery in order to identify those having MCI-PD.

### Diagnosis of Dementia

PDD was diagnosed according to the Movement Disorder Society criteria [19] based on neuropsychological examination and a clinical interview of the patient and a family member or friend, focusing on functional impairment due to cognitive decline.

### MCI-PD definition

Italian partners utilized the Level II (comprehensive assessment) operational schema according to MDS criteria [7]. Raw individual test values were converted into Z-scores using published Italian normative data corrected for age and education. PD patients performing ≤2 SD z-score below the population mean in at least two tests for the same cognitive domain or one test in at least two different domains were classified as MCI-PD (see Table 2 for cognitive outcomes). For the Czech cohort, a step-wise approach described above as recommended for the Level I (abbreviated assessment) was used [7].

### MRI Sequences

Whole head scans were acquired at the Czech centre (1.5T Siemens Symphony; T1 MPRAGE IR/GR sequence, TR 1700 ms, TE 3.93 ms, TI 1100 ms, flip angle 15°, 160 sagittal slices, voxel size 1.17×0.48×0.48 mm, FOV 246×246 mm, in plane matrix size 512×512) and at the Italian centre (1.5T Phillips Achieva; 3D TFE sequence, TR 1700 ms, TE 3.50 ms, flip angle 8°, 280 sagittal slices, voxel size 0.60×1.04×1.04 mm, FOV 250×250 mm, in plane matrix size 240×240).

### Image Preprocessing

All images were combined into one cohort and processed in the Czech centre (by RM) using SPM8 software (http://www.fil.ion.ucl.ac.uk/spm) with its internal toolbox DARTEL. Data were segmented into grey and white matter segments and registered using DARTEL to the study specific template. Resulting grey matter volume images were resampled to the resolution of 1.5×1.5×1.5 mm and smoothed with 8 mm FWHM Gaussian kernel.

### Statistical Analysis: SBM

The method uses spatial Independent Component Analysis (ICA) to reveal naturally grouping spatially independent sources of local grey matter volume variability with common co-variation among subjects. Initially the grey matter images were concatenated and reshaped to form 2D matrix with number of voxels and number of subjects as matrix dimension. Prior to ICA, Principal Components Analysis (PCA) was done to reduce the data dimensionality with the Minimum Description Length algorithm in order to estimate the optimal number of components [20]. These reduced data were then subject to spatial ICA using Infomax algorithm which decomposed the data into a source and a mixing matrix. The individual rows of the source matrix were reshaped back to the original 3D dimension and normalized to unit standard deviation. Such images depict the spatial characteristics of components, i.e. the sources of local grey matter volume variability. The columns of the mixing matrix could be seen as expressions which represent how much grey matter do individual subjects have in corresponding sources. This metric after filtering out the effect of age, gender, education, data origin (Italian and Czech centres) and total intracranial volume was subject to inter-group comparison. We employed a non-parametric Kruskall-Wallis (KW) test to identify those components with a significant group effect. Mann-Whitney post-hoc tests were used to identify significant differences between pairs of groups. The multiple testing problem was handled by False Discovery Rate (FDR) and the significance level was set to p<0.05. The images of significant components were spatially normalized to MNI space and threshold set at |Z|>3.0. The clusters with 100 and more adjacent voxels (>0.33 cm^3^) were superimposed on the study-specific grey matter images to visualize regions with strong inter-group differences.

### Correlation of MRI results with behavioural data

Pearson correlation analyses were used in order to assess relationship between components-of-interest expressions and cognitive tests Z-scores in the Italian cohorts. The multiple testing problem was handled by FDR and the significance level was set to p<0.05.

## Results

### Demographic and cognitive results

Subjects' characteristics are presented in Table 1. We found significant effects with regard to age (Kruskal-Wallis test [H_(3,N = 151)_ = 16.2, p = 0.001]) and education (Kruskal-Wallis test [H_(3,N = 151)_ = 14.3, p = 0.003]) between cognitive groups, but not for gender (Pearson chi-square test, p = 0.73). Detailed cognitive tests z-scores (Italian centre) and ACE-R domains z-scores (Czech centre) for all groups are displayed in Table 2. Eighteen out of 29 PD-MCI had single domain and 11 presented with multiple domain impairment.

### MRI results

**Figure 1:**
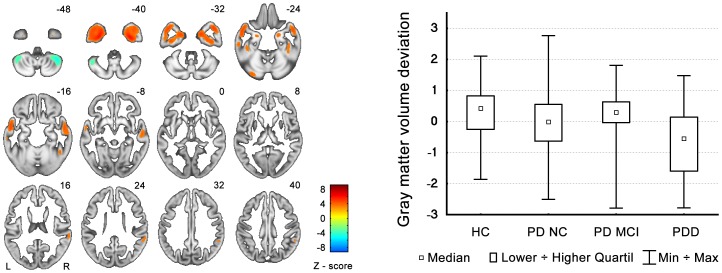
C1 component pattern and GMV changes in individual groups. The graph shows the deviations from the all-participants' mean. GMV decreases are depicted by red color and GMV increases are in green. The box graph on the right side shows median GMV changes in individual groups of subjects.

**Figure 2:**
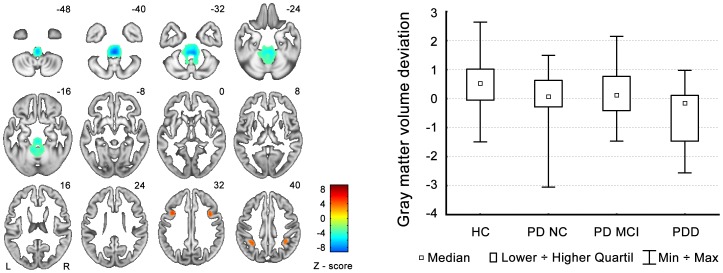
C2 component pattern and GMV changes in individual groups. The graph shows the deviations from the all-participants' mean. GMV decreases are depicted by red color and GMV increases are in green. The box graph on the right side shows median GMV changes in individual groups of subjects.

Out of 14 independent components, there was a significant group effect in two components. The first one (C1; Kruskal-Wallis test H_(3,N = 151) = _12.4, p = 0.0063) represented areas of GMV reduction particularly in the bilateral temporal lobes including the inferior temporal gyrus, hippocampus, parahippocampal gyrus, amygdala, fusiform gyrus, and medial and superior temporal gyri, as well as areas of GMV increases in the posterior lobes of both cerebellar hemispheres (see Figure 1 and Table 3). The post-hoc analysis revealed significant differences only between the PDD group and all other groups (see the box graph in Figure 1). The second component (C2; Kruskal-Wallis test H_(3,N = 151) = _13.2, p = 0.0042) represented areas of bilateral grey matter reduction in the medial frontal and inferior parietal regions, as well as areas of GMV increases in the brainstem and cerebellum (see Figure 2 and Table 3). The post-hoc analysis again revealed significant differences between the PDD group and all other groups. Unlike in the case of the C1 component, significant differences were already present between the PD-NC group and the HC group (see the box graph in Figure 2). However, we were not able to differentiate PD-MCI from PD-NC.

**Table 3:** Patterns of GMV changes.

Region	Side	MNI coordinates [mm]	Number of voxels	Z-value in maximum
**C1 COMPONENT**				
**GMV decreases**				
Inferior temporal gyrus, hippocampus, parahippocampal gyrus, amygdala, fusiform gyrus, middle temporal gyrus, superior temporal gyrus	L	x = −32, y = −4, z = −41	5 900	5.87
Inferior temporal gyrus, parahippocampal gyrus, superior temporal gyrus	R	x = 32, y = −6, z = −40	5 402	5.84
Cerebellum – declive	L	x = −32, y = −82, z = −25	268	3.84
Superior temporal gyrus, supramarginal gyrus	R	x = 63, y = −39, z = 21	427	3.67
**GMV increases**				
Cerebellum	L	x = −42, y = −54, z = −44	692	−4.49
Cerebellum	R	x = 42, y = −52, z = −47	775	−5.02
**C2 COMPONENT**				
**GMV decreases**				
Middle frontal gyrus	L	x = −38, y = 13, z = 31	298	5.21
Inferior parietal lobule	R	x = −32, y = −49, z = 43	401	5.59
Middle frontal gyrus	L	x = 39, y = 10, z = 33	255	4.59
Inferior parietal lobule	R	x = 36, y = −46, z = 43	515	4.35
**GMV increases**				
Pons, midbrain, cerebellum	L	x = 2, y = −37, z = −40	9 930	−9.41

### Association between MRI component expressions and cognitive results in the Italian PDD group

**Figure 3:**
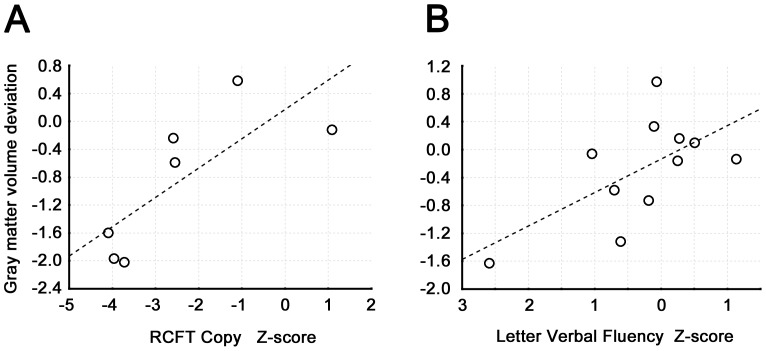
Association between component expressions and cognitive results. Fig. 3A shows correlation between the C1 component loadings and Rey-Osterrieth Complex Figure Test copy z-scores; Fig. 3B displays correlation between C2 component loadings and Letter Verbal Fluency Task z-scores.

We observed significant association between the C1 component loadings and impairment in visuo-spatial functions as assessed by the Rey-Osterrieth Complex Figure Test (RCFT) copy (R = 0.63, p = 0.02, N = 11; see Figure 3A) and between the C2 component loadings and the phonemic fluency task (R = 0.77, p = 0.009, N = 7; see Figure 3B). We did not observe any significant associations between individual cognitive test outcomes and C1/C2 loadings in the MCI-PD group or in the PD-NC group.

## Discussion

We are reporting results from a two-centre study using for the first time SBM in order to evaluate specific differences in GMV patterns across 4 groups of subjects: HC, PD-NC, MCI-PD and PDD. In previous studies, high variability of brain morphology has been encountered in PD patients with cognitive impairment, including grey matter reduction particularly in fronto-temporal and posterior cortical areas [5], [21]–[28]. Most previous studies have employed mass-univariate techniques - that is, the image of the brain is divided into basic units of volume (voxels) and a statistical analysis is performed for every individual voxel, without taking into account the information regarding its surroundings. The utility of mass-univariate approaches has been questioned several times - the issues of sensitivity or the ability to correctly characterize multivariate brain morphology have been raised [29]. Unlike the VBM, the SBM is a model-free exploratory method looking into the whole brain and searching for specific GMV patterns that explain the independent sources of GMV variability across groups.

In the present study we were able to detect two independent patterns of GMV changes. The first (C1) involved particularly the GMV reductions in the hippocampus, amygdala and neocortical temporal regions, while the second (C2) involved GMV major deviations in the fronto-parietal regions and the pons/cerebellum. The temporal lobe atrophy pattern was related to impairment in visuo-spatial abilities in PDD, which was in fact the most affected cognitive domain in this group. The inferior and medial temporal cortex is the final route of the ventral visual pathway [30], and our observation fits well with the results of previous studies reporting hippocampal atrophy in PD patients at different stages of cognitive decline [31]–[32], as well as an association between visuospatial tasks and GMV in medial temporal cortex in PD [33]. It has been hypothesized that posterior temporo-parietal changes are caused by structural pathology such as Lewy body and amyloid deposition and induce aggressive course of the disease particularly with regard to cognitive decline [34], [35].

Our C2 pattern consists of both GMV decreases in the middle frontal gyrus and inferior parietal lobules including the supramarginal gyrus (i.e. areas involved in the ventral attention network) [36], and GMV increases in the infratentorial regions of the pons and cerebellum. It mirrors the previously described metabolic cognitive pattern in PD [37]. Cognitive role of the cerebellum, pons and of the fronto-parieto-ponto-cerebellar networks has been documented [38], [39]. The ventral attention network is recruited by tasks such as orienting to unexpected but relevant stimuli [40], and changes within this network may underlie the characteristic impairment of attentional mechanisms in PD [41].

The C2 pattern differentiated PDD from all other groups and was associated with impairment in letter verbal fluency task. In addition, the C2 pattern differentiated PD-NC from HC. Of note, HC participants were significantly better educated than the PC-MCI subjects which might indicate a higher level of cognitive reserve in this patient group [42], and might theoretically explain the fact that our MCI-PD participants did not significantly differ from HC in their C2 pattern expression.

To our knowledge this is the first time that metabolic changes assessed by the FDG-PET are replicated in a structural MRI study. In that study the authors differentiated multiple-domain PD-MCI from PD-NC but not single-domain PD-MCI from PD-NC [37]. However, their definition of MCI was different. Moreover, the majority of our PD-MCI patients had single-domain impairment and thus were possibly at an early stage of cognitive decline compared to those with multi-domain MCI. Our result in PD-MCI is also in line with other recent MRI studies performed at a very early stage of cognitive decline and applying rather comprehensive neuropsychological assessment for MCI diagnosis [4]–[6].

While it is well accepted that regional metabolic decreases and increases can coexist in PD, it may be more difficult to explain the GMV increases observed in the infratentorial regions. Unfortunately, underlying mechanisms for these changes cannot be directly answered and resolved by structural MRI.

Using fMRI, several studies have shown that L-dopa may modify (increase) connectivity of specific brain networks [16], including those with a major engagement of cerebellar and brainstem regions [43]. Moreover, there is some emerging evidence suggesting that L-dopa treatment may lead to GMV increases as well. Recently, such GMV increases were reported in bilateral inferior frontal gyri in PD patients with levodopa-induced dyskinesias (LIDs) as compared to those without LIDs [44]. The authors hypothesized that levodopa applied in a pulsatile and non-physiological manner could affect the normal physiological mechanisms that mediate motor control and result in aberrant neural plasticity. However, in our study the levodopa equivalent dose was not correlated with our GMV patterns in the PDD group (R = 0.14 for C1 and 0.13 for the C2 component). It has also been speculated that neuroplasticity following years of increased use of executive control to override involuntary movements might be involved in patients with LIDs [45]. However, since our patients did not have LIDs, this is an unlikely explanation of our findings.

Our aim was to investigate GMV deviations associated with cognitive decline in PD and in this regard results of a recent study by Biundo et al. [46] are more relevant to our findings. The authors have shown that both cortical thinning and thickening can co-exist in cognitively impaired PD patients and probably reflect compensatory plasticity within specific networks.

Here we show for the first time that both deficits and compensatory mechanisms may be involved in PD patients who do not yet meet the criteria for PD-MCI. Interestingly, these patterns become more pronounced at later stages of cognitive decline – when dementia develops. Although this is a novel morphological result in PD population it accords well also with functional results described in MCI-AD and AD-dementia patients [47]. The authors found both decreased and increased connectivity within the default mode network in their MCI-AD while this pattern was even more pronounced in AD patients at clearly established dementia stages. Since the engagement of component patterns in the present study correlated with cognitive test results in PDD it is possible that increases of GMV within our components reflect malfunction related to brain pathology, or compensation that is not efficient enough to counterbalance cognitive impairment, or leading to cognitive dysfunction per se.

Finally, it has to be acknowledged that both grey matter and white matter tracts are present in the pons as well as in the cerebellum. Therefore, our result of increased GMV in these regions should be considered with care, as different microanatomical factors could be related to described changes [48].

We have to acknowledge our study has limitations. Even if both centres followed operational guidelines to diagnose MCI-PD we applied different instruments for cognitive testing. The respective groups of participants (HC, PD-NC, PD-MCI, PDD) differed with respect to their age and years of education. We controlled for these variables in the second-level analysis when comparing the groups. Nevertheless, the possible effects of age and length of education cannot be fully excluded. We also have to acknowledge an unbalance in the number of participants in specific subgroups (75 PD-CN vs. 25 HC, 29 MCI-PD and 22 PDD). This discrepancy in sample sizes could have lowered the power of the Kruskal-Wallis test.

We report correlations between the MRI component loadings and specific cognitive outcomes in the Italian PDD groups which are small in size. Although the correlations support our hypothesis the results cannot be generalized. The strength of our study include comparison of a fairly large number of controls and PD patients at well-defined stages of cognitive decline, application of the novel consensus criteria for PD-MCI along with a comprehensive neuropsychological battery for the largest centre. Finally, our SBM analysis is a novel method allowing identification of patterns of grey-matter loss which may more closely mirror the actual changes occurring in PD compared to traditional univariate voxelwise analyses.

Future prospective studies will be required to investigate whether these structural patterns can be used as biomarker of cognitive progression.
